# Therapeutic efficacy of neuregulin 1-expressing human adipose-derived mesenchymal stem cells for ischemic stroke

**DOI:** 10.1371/journal.pone.0222587

**Published:** 2019-09-27

**Authors:** Sun Ryu, Jae-Min Lee, Cheong A. Bae, Chae-Eun Moon, Kyung-Ok Cho

**Affiliations:** 1 Department of Pharmacology, College of Medicine, The Catholic University of Korea, Seoul, South Korea; 2 Catholic Neuroscience Institute, College of Medicine, The Catholic University of Korea, Seoul, South Korea; 3 Department of Biomedicine & Health Sciences, College of Medicine, The Catholic University of Korea, Seoul, South Korea; 4 Institute of Aging and Metabolic Diseases, College of Medicine, The Catholic University of Korea, Seoul, South Korea; Universita degli Studi di Napoli Federico II, ITALY

## Abstract

Adipose-derived mesenchymal stem cells (AdMSCs) have been reported to ameliorate neurological deficits after acute ischemic stroke. As neuregulin 1 (NRG1, or heregulin 1), a growth factor with versatile functions in the central nervous system, has demonstrated protective effects against ischemic brain injuries, we have generated NRG1-overexpressing AdMSCs in order to investigate whether NRG1-AdMSCs could enhance therapeutic benefits of AdMSCs in ischemic stroke. After AdMSCs were infected with adenoviral NRG1, increased NRG1 secretion in NRG1-AdMSCs was confirmed with ELISA. At 1 d after ischemic stroke that was induced by the occlusion of middle cerebral artery (MCAo) for 60 min in Sprague Dawley (SD) rats, adenoviral NRG1, AdMSCs, NRG1-AdMSCs, or PBS were injected into the striatum and serial neurologic examinations were performed. Administration of NRG1-AdMSCs resulted in significant improvement of functional outcome following stroke compared to AdMSCs- or adenoviral NRG1-treated group, in addition to the reduction in the infarct size evaluated by hematoxylin and eosin staining. When NRG1 expression in the brain was examined by double immunofluorescence to human nuclei (HuNu)/NRG1 and ELISA, NRG1-AdMSCs demonstrated marked increase in NRG1 expression. Moreover, western blot analysis further showed that transplantation of NRG1-AdMSCs significantly increased both endogenous and adenoviral NRG1 expression compared to AdMSCs-treated group. To elucidate molecular mechanisms, NRG1-associated downstream molecules were evaluated by western blot analysis. Expression of ErbB4, a receptor for NRG1, was markedly increased by NRG1-AdMSCs administration, in addition to pMAPK and pAkt, crucial molecules of NRG1-ErbB4 signaling. Taken together, our data suggest that NRG1-AdMSCs can provide excellent therapeutic potential in ischemic stroke by activating NRG1-ErbB4 signaling network.

## Introduction

Stroke is an acute cerebrovascular disorder caused by insufficient blood supply to the brain due to ischemia or hemorrhage, resulting in extensive loss of neurons and their connections in the damaged brain [1]. Current approved therapies for ischemic stroke are restricted to fast recanalization of vascular obstruction and pharmacologic administration of thrombolytic agents, trying to remove inciting stimuli [2]. New approaches including cell-based therapy with multipotent stem cells have demonstrated great promises for functional recovery in patients with stroke [3, 4]. In particular, mesenchymal stem cells (MSCs) have shown substantial benefits due to their immunosuppressive properties and low immunogenicity [5]. Among various MSCs, adipose-derived mesenchymal stem cells (AdMSCs) show several advantages in clinical applications for stroke [6]. Considering abundance of adipose tissues, AdMSCs can be easily obtained by liposuction. Moreover, AdMSCs can proliferate and differentiate into neurons without significant adverse effects [7, 8], thereby alleviating ethical concerns. However, even although stem cell therapy is proven to be feasible for stroke [9] and vigorous research efforts have elucidated details of MSCs behaviors after brain injury, MSCs transplantation has achieved only modest success for the treatment of stroke. Therefore, it is no doubt that innovative approaches or functional improvements of MSCs are required to enhance therapeutic efficacy of current MSCs transplantation.

Neuregulin 1 (NRG1) is a trophic factor encoded by the NRG1 gene that can be alternatively spliced into six proteins (I–VI); these isoforms include Neu differentiation factor, heregulin, acetylcholine receptor inducing activity, glial growth factor, and sensory and motor neuron-derived factor [10–13]. All NRG1 isoforms contain an epidermal growth factor (EGF)-like domain and the majorities of NRG1 isotypes are transmembrane protein that requires additional proteolytic cleavage to produce a paracrine signal [10, 14, 15]. Once secreted, NRG1 can bind ErbB receptor tyrosine kinases, initiating receptor dimerization for the activation of downstream signaling [16–18]. NRG1 and ErbB tyrosine kinases are widely expressed in the brain, playing key roles in the regulation of neuronal survival, migration of precursor cells, and the expression of neurotransmitter receptors and ion channels [10, 18, 19]. Moreover, multiple brain diseases including multiple sclerosis, traumatic brain injury, and stroke can enhance NRG1-ErbB signaling networks [20–23]. NRG1 administration itself could improve neurological outcome in ischemic stroke [24, 25], implying additional therapeutic effects if MSCs can produce NRG1 more efficiently. In addition, as NRG1 could suppress stroke-induced pro-inflammatory and stress-related gene expressions, which are related with the molecular mechanisms of MSCs in ischemic stroke [24, 26], NRG1 can be an attractive target for MSCs modification to maximize therapeutic efficacies of cell-based therapy.

Therefore, we engineered AdMSCs to boost NRG1 secretion by adenoviral infection and investigated whether these NRG1-modified AdMSCs (NRG1-AdMSCs) could have better therapeutic potentials after ischemic stroke in rats. We found that transplantation of NRG1-AdMSCs could promote functional recovery with the reduction of infarct volume, compared to adenoviral NRG1-, AdMSCs- or PBS-injected groups. Moreover, NRG1-AdMSCs increased the expression of adenoviral and endogenous NRG1 expression in the ischemic hemisphere. Finally, NRG1-AdMSCs upregulated ipsilateral expressions of ErbB4, phosphorylated mitogen-activated protein kinase (pMAPK), and pAkt, suggesting NRG1-mediated activation of ErbB4 downstream signaling. Together, these findings highlight therapeutic benefits of NRG1-AdMSCs in ischemic stroke, possibly through the stimulation of NRG1-ErbB4 signaling networks.

## Materials and methods

### Preparation and adenoviral infection of AdMSCs

Human mesenchymal stem cells were isolated from waste adipose tissues during surgery after obtaining the written informed consent with the approval of the Institutional Review Board of Catholic University of Korea (Approval number: KC11TNSI0186). At passage 4, AdMSCs were grown in low glucose Dulbecco’s modified Eagle’s medium (DMEM) (Hyclon, GE Healthcare Life Sciences, UK) supplemented with 10% fetal bovine serum (FBS), penicillin and streptomycin at 37°C. Adenovirus overexpressing NRG1 was produced as previously described [27]. For adenoviral infection, AdMSCs were incubated with adenovirus expressing a secreted form of EGF-like domain from NRG1β at a multiplicity of infection (MOI) of 100 in 2% FBS-containing media for 2 h [27]. Medium was then replaced with fresh DMEM containing 10% FBS. AdMSCs were transplanted into the rat brain after 24 h of additional culture.

### Rat model of transient focal cerebral ischemia and injection of adenoviral NRG1, AdMSCs or NRG1-AdMSCs

Specific pathogen-free 6-week-old male Sprague-Dawley (SD) rats were obtained from the Orient Bio (Seoul, Korea) and used after 2 weeks of quarantine and acclimatization. The animals were housed in a room maintained at 22 ± 3°C under a relative humidity of 50 ± 10% with artificial lighting from 08:00 to 20:00. The animals were kept in individually ventilated plastic cages with wire mesh tops and allowed sterilized tap water and commercial rodent chow (TD2018S, Harlan Laboratories, USA) *ad libitum*. Animal health monitoring was performed twice a year using sentinel animals to check pathogenic viruses, bacteria, and protozoans. The vivarium was accredited by the Korea Excellence Animal Laboratory Facility from Korea Food and Drug Administration in 2017 and the Association for the Assessment and Accreditation of Laboratory Animal Care International (AAALAC International) in 2018. All surgical interventions and presurgical and postsurgical animal care were provided in accordance with the Laboratory Animals Welfare, the Guide for the Care and Use of Laboratory Animals of the National Institute of Health. The protocol was approved by the Institutional Animal Care and Use Committee (IACUC) in School of Medicine, The Catholic University of Korea (Approval number: CUMS-2018-0089-01). An effort was made to minimize the pain and suffering of the animals such as buprenorphine administration when needed. Transient focal cerebral ischemia was induced by the intraluminal occlusion of the left middle cerebral artery (MCAo) as previously described with minor modifications [28–30]. Briefly, male SD rats, weighing 270–300 g, were anesthetized with 1.5% isoflurane in a 70% nitrous oxide and 30% oxygen mixture using a face mask. After midline neck incision, a 5–0 silicon-coated nylon monofilament (Doccol Corporation, USA) was inserted through the external carotid artery and into the internal carotid artery. At 1 h after the occlusion of the left MCA, the filament was withdrawn to allow reperfusion and animals were placed in the incubator. During cerebral ischemia, rectal temperature was maintained at 37 ± 0.5°C using a thermistor-controlled heating blanket. At one day after MCAo, rats were reanesthetized with ketamine (100 mg/kg) and xylazine (10 mg/kg) cocktails and placed in a stereotaxic frame (David Kopf instruments, USA) for adenoviral NRG1 or AdMSCs injection. After a burr hole was made with a stereotaxic-attached drill, 2 μL of adenoviral NRG1 (1 × 10^10^ pfu/ml) or 5 μL of 1.2 × 10^5^ AdMSCs/μL in phosphate-buffered saline (PBS) were injected with a Hamilton syringe into the left lateral striatum (0.7 mm anterior to the bregma, 3.2 mm lateral to the midline, and 5.5 mm beneath the dura) for 5 min, and the needle was left for an additional 5 min after the injection. Control group was injected with PBS instead of stem cells. Subsequently, dental cement was applied on the skull and the animals were returned to their cages once they become conscious. During the post-stroke care, animals were humanely euthanized with an overdose of ketamine and xylazine cocktails when paralysis, dysbasia, dyschezia, or the weight loss greater than 20% at 7 d after MCAo was observed. We did not find any mortality that occurred outside of planned euthanasia and humane endpoints we set.

### Behavioral assessment

One of the most common neurological scales used in animal studies of stroke is the modified Neurologic Severity Score (mNSS), which can assess motor (muscle tone and abnormal movement, maximum 6 points), sensory (visual, tactile, and proprioceptive, maximum 2 points), reflex (pinna, corneal, startle, and myoclonus or myodystony, maximum 4 points), limb placement test (maximum 10 points), and balance tests (maximum 6 points) [31]. Using this mNSS system with minor modification, we gave scores to the rats when they showed functional deficits, grading the test results from 0 (normal) to 28 (maximal deficit score). In the morning at 1 d after MCAo, rats showing lower than 23 points in the mNSS test were excluded due to insufficient behavioral deficits (25 out of 126 rats) [31]. Only rats qualifying this criterion were further evaluated by mNSS at 7, and 14 d after MCAo. After the behavioral test was completed, some animals were perfused for the staining and the rest of the animals were sacrificed for western blotting and enzyme-linked immunosorbent assay (ELISA).

### Measurement of infarct sizes

At 14 d after MCAo, animals were fully anesthetized with an overdose of ketamine and xylazine cocktails. After cardiac perfusion with normal saline and fixation with 4% paraformaldehyde in 0.1 M PBS, the brains were removed and cryoprotected with 30% sucrose solution. Then, the brains were cut into 40-μm-thick coronal sections by using a cryostat microtome. Total of six sections with 2 mm intervals between each section were processed for hematoxylin and eosin (H&E) staining. Briefly, sections were stained in hematoxylin solution for 10 min, followed by washing with tap water. Then the sections were incubated with Eosin for 10 s, dehydrated, and mounted. Infarct region was defined as the area with eosinophilic neurons showing shrunken cell bodies, dark pyknotic nuclei, and the intense red cytoplasm [30, 32]. Using an image analysis program (Image-Pro Plus, USA), infarct area was measured by subtracting the area of the ipsilateral non-infarcted hemisphere from that of the contralateral tissue. The percentage of infarct volume was calculated by dividing the sum of the infarct areas by the areas of contralateral hemisphere.

### Enzyme-linked immunosorbent assay (ELISA)

The secretion of adenoviral NRG1 into culture media and the brain NRG1 level after MCAo were measured using a commercial NRG1-β1 ELISA system (R&D Systems, USA). 3 × 10^5^ AdMSCs in a 35 mm culture dish were infected with NRG1-expressing adenovirus at 100 MOI in 2% FBS containing media for 2 h. Medium was replaced with fresh DMEM containing 10% FBS and the cells were cultured for an additional 12 h. Media was then removed and the cells were incubated for 48 h in 1 ml of serum-free DMEM. The culture supernatants were collected and stored at -70°C until analysis. Then, ELISA analysis was performed according to the manufacturer’s instruction.

With regard to brain NRG1 expression, coronal brain tissue chunks (–1.0 ~ 1.0 mm to the bregma) were collected at 14 d after MCAO. Ischemic hemispheres (200 mg) were dissected on ice and were stored at -70°C until use. Subsequently, each tissue sample was resuspended in an equal weight of homogenization buffer and homogenized with a Dounce homogenizer. The homogenate was centrifuged (10,000 g) for 10 min at 4°C and the supernatant (5 μg/μl) was processed for ELISA analysis.

### Staining

For immunocytochemistry, AdMSCs and NRG1-AdMSCs were grown in four-well Lab-Tek II chamber slides (Nunc, USA) at a density of 2×10^4^ cells/mL. Briefly, the cells were fixed with 4% paraformaldehyde for 30 min at 4°C. The cells were incubated in blocking solution (10% normal donkey serum) for 30 min and then with rabbit polyclonal anti-NRG1 (1:200; Santa Cruz Biotechnology, USA) for 16 h at 4°C. After washing with PBS, the cells were incubated with Cy3-conjugated donkey anti-mouse IgG (1:500; Jackson ImmunoResearch, USA) for 1 h at room temperature (22 ± 3°C). After final washing with PBS, the cells were counterstained with 4,6-diamidino-2-phenylindole (DAPI).

For immunohistochemical staining, the 40-μm-thick coronal sections were incubated with 10% normal donkey serum for 1 h and then with the following primary antibodies for 16 h at 4°C: rabbit polyclonal anti-NRG1 (1:200; Santa Cruz Biotechnology), mouse monoclonal anti-human nuclei (1:200; Millipore Corporation, USA). After washing with PBS, the sections were incubated with the following secondary antibodies for 2 h at room temperature (22 ± 3°C): Cy3-conjugated donkey anti-mouse IgG (1:500; Jackson ImmunoResearch), Alexa488-conjugated donkey anti-rabbit IgG (1:500; Jackson ImmunoResearch). Then, after washing with PBS, the sections were mounted and observed using a confocal microscopy (LSM 510 Meta; Carl Zeiss Co., Germany).

### Western blotting

Brain tissues were lysed in RIPA buffer (25 mM Tris-Hcl (pH 7.6), 150 mM NaCl, 1% NP-40, 1% sodium deoxycholate, and 0.1% SDS) added with protease and phosphatase inhibitor cocktail (ThermoFisher Scientific, USA). Lysates were clarified by centrifugation and the supernatant was collected for protein extraction. Equal amounts of protein (60 μg) from each sample were separated on 8~15% SDS-polyacrylamide gels and transferred to nitrocellulose membranes. The membrane was then blocked with 5% bovine serum albumin in tris-buffered saline containing 0.1% Tween-20 for 1 h at room temperature and incubated overnight at 4°C with anti-Neuregulin-1 (1:500, Santa Cruz Biotechnology), anti-ErbB4 (1:500, Santa Cruz Biotechnology), p44/42 mitogen-activated protein kinase (MAPK; Cell signaling Technology, USA), anti-phospho MAPK (Cell signaling Technology), anti-Akt (Cell signaling Technology), or anti-phospho Akt (Cell signaling Technology) antibodies diluted 1:1000. The membrane was then incubated with horseradish peroxidase-conjugated secondary anti-rabbit, mouse antibody (1:1000, Santa Cruz Biotechnology) at room temperature for 1 h. Bands were detected using an enhanced chemiluminescence detection kit (Thermo Scientific, USA), after which these blots were re-probed with mouse monoclonal anti-GAPDH antibody (1:1000; Novus) as a loading control for all experiments. Relative quantification of the band immunoreactivity was performed by densitometric analysis using an ImageQuant LAS 4000 (Fujifilm, Japan).

### Statistical analysis

The data are presented as the mean ± SEM. No statistical methods were used to determine sample sizes, but our sample sizes are similar to those reported in previous publications [9, 23, 24, 33, 34]. Statistical significance was assessed using SPSS software (version 21.0, IBM SPSS Corp.) for mNSS and western blot analysis, and GraphPad Prism 7 software (GraphPad Software Inc., USA) for the rest of statistical analysis. Student’s unpaired t-test was performed to compare *in vitro* ELISA, and NRG1 expression between the groups as the data passed Shapiro-Wilk normality test. As for behavioral test, repeated measures ANOVA was performed and for mNSS comparison at 14 d after stroke, one-way ANOVA followed by Tukey’s post hoc test was carried out. In addition, infarct volume was assessed by Kruskal-Wallis test and uncorrected Dunn’s post hoc test as the data failed to pass the normality test. Finally, *in vivo* ELISA and western blotting of downstream signaling were analyzed by one-way ANOVA followed by Duncan’s or LSD post hoc test, respectively. p < 0.05 was considered statistically significant.

## Results

### AdMSCs infected with NRG1-expressing adenovirus release NRG1 protein *in vitro*

**Fig 1:**
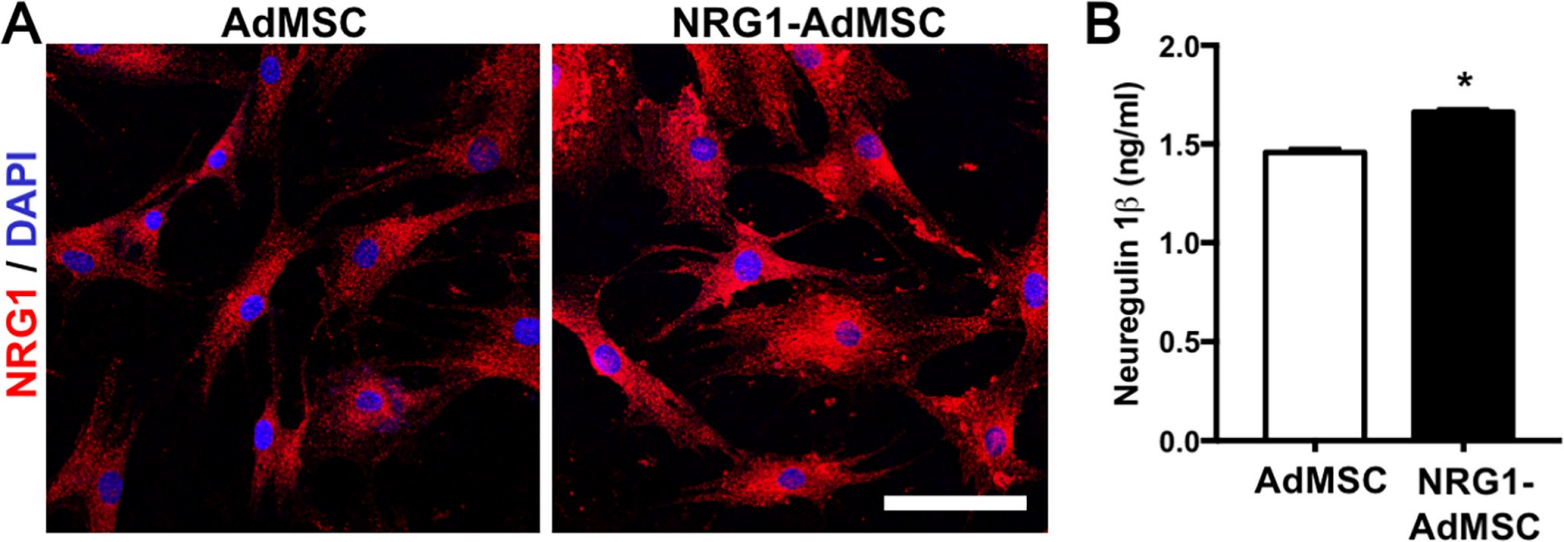
NRG1-expressing AdMSCs release NRG1 protein *in vitro*. (A) Immunocytochemistry to NRG1 shows NRG1 expression in both AdMSCs and NRG1-AdMSCs. Scale bar = 100 μm. (B) NRG1 secretion by AdMSCs and NRG1-AdMSCs is measured with ELISA (n = 4 per group). NRG1 release into the media is significantly increased in NRG1-expressing AdMSCs. *p < 0.05 by Student’s unpaired t-test. The data are presented as the mean ± SEM.

After we infected AdMSCs with NRG1-expressing adenovirus, we evaluated NRG1 expression with immunocytochemistry. NRG1 immunoreactivity was shown in both AdMSCs treated with or without adenovirus (Fig 1A). Since the adenovirus used in this study contained a secreted form of EGF-like domain from NRG1β, we performed quantitative analysis of NRG1 secretion using ELISA (Fig 1B). Higher levels of NRG1 protein was detected in the media collected from NRG1-AdMSCs, compared to that from control AdMSCs (t(6) = 7.81, p < 0.01, n = 4 per group). Taken together, these findings demonstrated that AdMSCs engineered to produce NRG1 could have a greater capacity to release NRG1, although AdMSCs themselves could secrete low levels of NRG1.

### NRG1-expressing AdMSCs improve neurological function with the reduction of infarct volume after ischemic stroke

**Fig 2:**
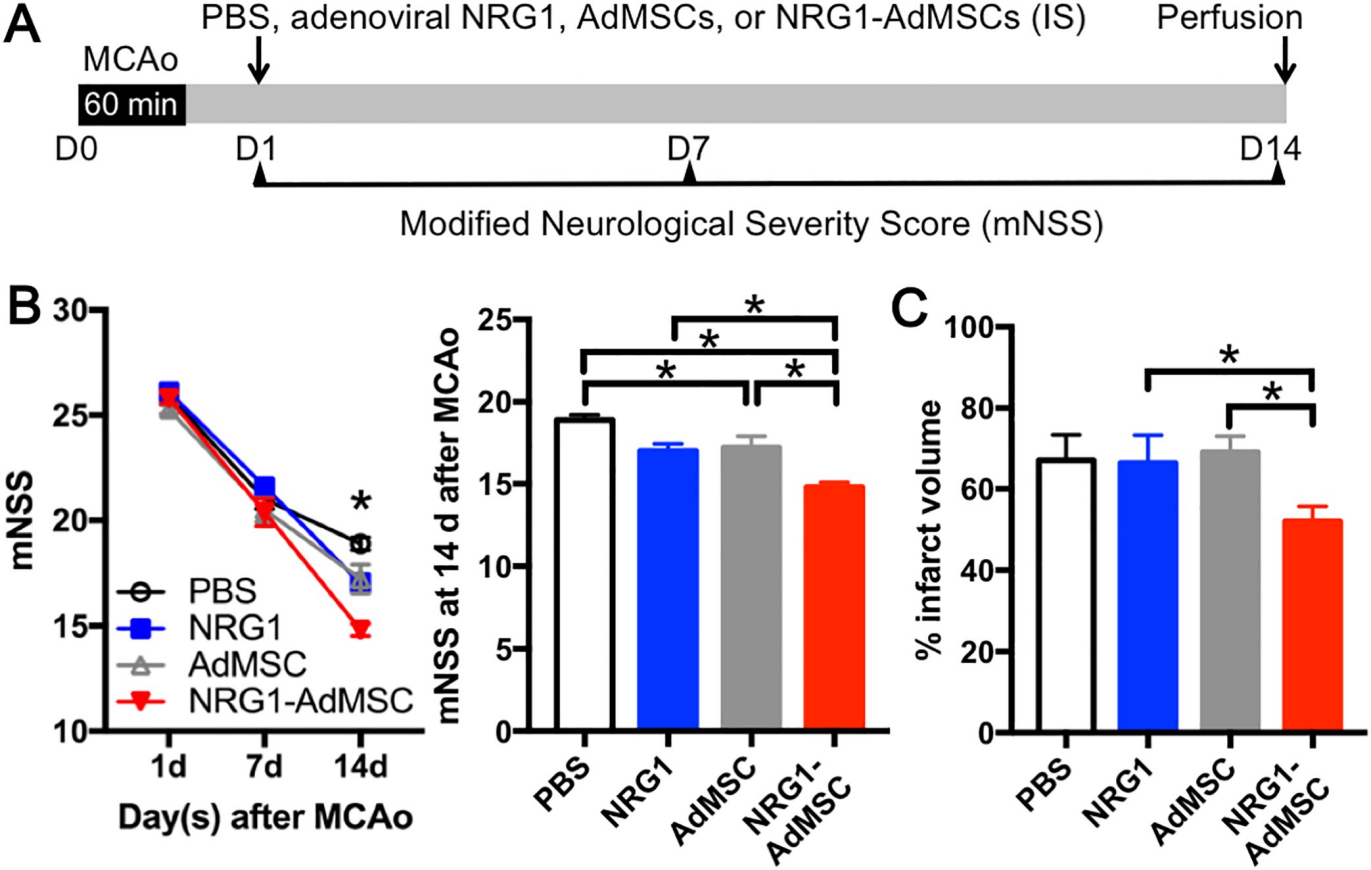
NRG1-expressing AdMSCs enhance neurological functional recovery and reduce infarct volume. (A) Experimental design for ischemic stroke and MSC transplantation. (B) mNSS is performed at 1, 7, and 14 days after MCAo. Rats treated with NRG1-AdMSCs (n = 26) show significantly improved neurological function compared to PBS- (n = 19), adenoviral NRG1- (n = 10), or AdMSCs-treated rats (n = 18). *p < 0.05 by repeated measures ANOVA for the left graph. *p < 0.05 by one-way ANOVA, followed by Tukey’s post hoc test for the right graph. (C) Rats transplanted with NRG1-AdMSCs (n = 10) show a significant reduction in infarct volume compared to PBS- (n = 6), adenoviral NRG1- (n = 4), or AdMSCs-treated rats (n = 7) at 14 days after MCAo. *p < 0.05 by Kruskal-Wallis test followed by uncorrected Dunn’s test. The data are presented as the mean ± SEM.

We then assessed the functional impact of NRG1-AdMSCs transplantation after ischemic stroke using mNSS test. As we found that all of the MCAo-subjected animals showed comparable neurological deficits at 1 d after stroke with very low individual variations, we randomly assigned three groups for adenoviral NRG1, AdMSCs, NRG1-AdMSCs, or PBS injection and performed transplantation at 1 d post-stroke after we confirmed similar neurological outcomes by mNSS test. Then, motor function was further evaluated at 7 and 14 d post-stroke (Fig 2A). Temporal improvement of neurological function after MCAo was observed regardless of the groups (F2,138 = 418.06, p < 0.01; Fig 2B). However, rats transplanted with NRG1-AdMSCs (n = 26) demonstrated significant lower mNSS scores, compared to PBS- (n = 19), adenoviral NRG1 (n = 10), or AdMSCs-treated groups (n = 18) (F3,69 = 5.55, p < 0.01). When we further assessed neurological outcomes among 4 groups at 14 d after MCAo, NRG1-AdMSCs-treated rats showed greater functional recovery than adenoviral NRG1- or AdMSCs-treated group, suggesting excellent therapeutic efficacy of NRG1-expressing AdMSCs (Fig 2B). In support of mNSS results, infarct size was markedly reduced in rats transplanted with NRG1-AdMSCs (n = 10) compared to PBS-injected controls (n = 6), adenoviral NRG1- (n = 4) or AdMSCs-treated rats (n = 7) (H = 8.46, p = 0.04; Fig 2C).

### NRG1-expressing AdMSCs show an increased NRG1 expression in the ischemic hemisphere

**Fig 3:**
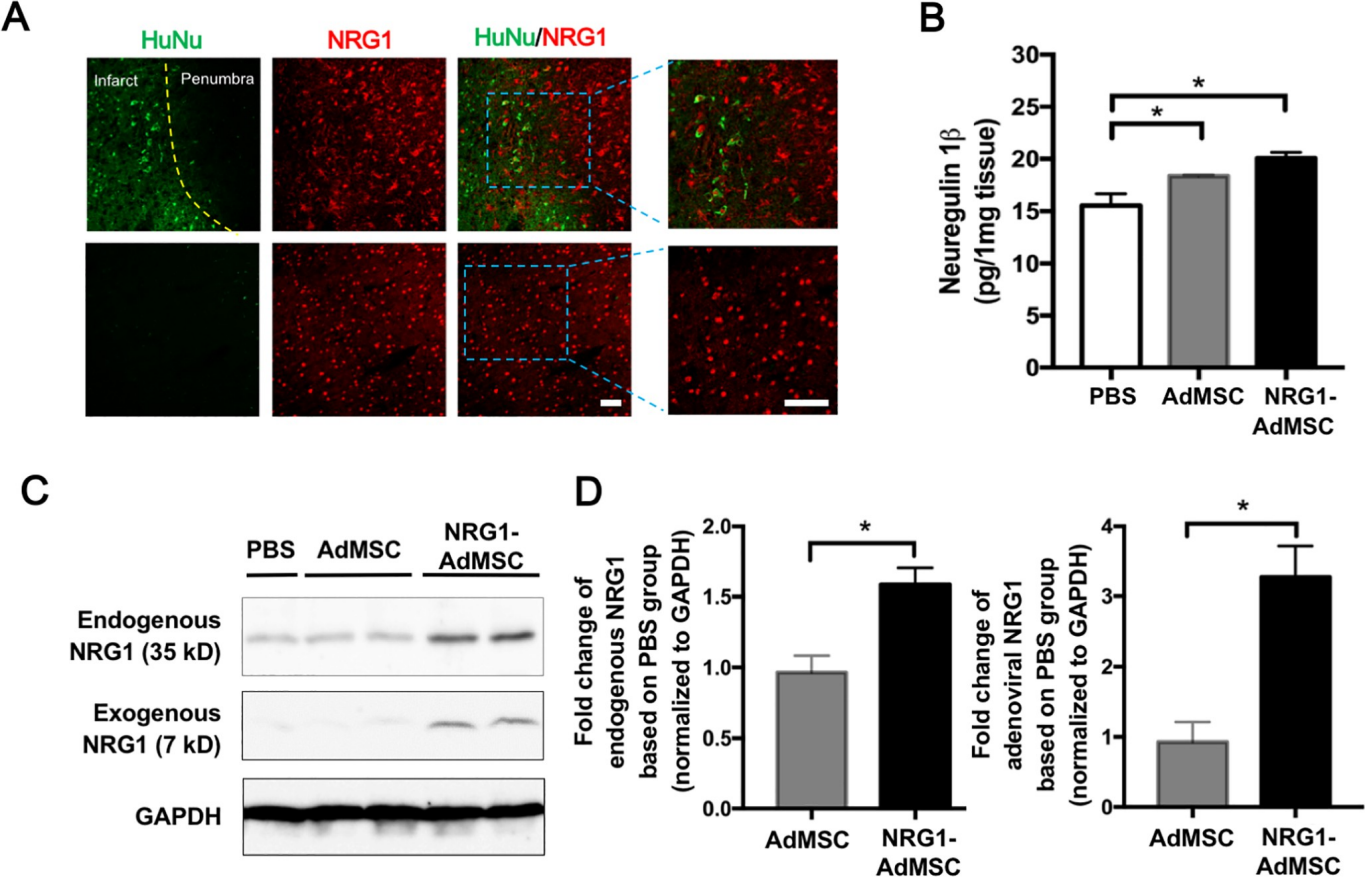
Transplantation of NRG1-expressing AdMSCs increases NRG1 expression in the ipsilateral hemisphere. (A) Immunohistochemical staining shows that NRG1 immunoreactivity (red) is co-localized with human nuclei (HuNu, green) in the ipsilateral hemisphere whereas no double-labeled cells are detected in the contralateral hemisphere. Scale bar in the low magnified image = 100 μm. Scale bar in the high magnified image = 100 μm. (B) Protein level of NRG1 determined by ELISA is significantly increased by NRG1-AdMSCs transplantation (n = 3 for each group). *p < 0.05 by one-way ANOVA followed by Duncan’s post hoc test. (C) Representative western blot of three independent experiments of exogenous and endogenous NRG1. (D) Graphs showing relative expression levels of endogenous and exogenous NRG1 between AdMSCs- (n = 4) and NRG1-AdMSCs- (n = 6) treated groups. Both endogenous NRG1 (35 kD) and exogenous adenoviral NRG (7 kD) are increased by the transplantation of NRG1-AdMSCs. *p < 0.05 by Student’s unpaired t-test. The data are presented as the mean ± SEM.

To determine NRG1 expression in the transplanted AdMSCs and NRG1-AdMSCs, we performed double immunofluorescence with antibodies specific to human nuclei (HuNu) and NRG1 (Fig 3A). HuNu-positive AdMSCs were mainly located in the infarct area co-expressing NRG1 (Fig 3A), consistent with AdMSCs and NRG1-AdMSCs *in vitro*. Meanwhile, there were no HuNu-labeled cells in the contralateral hemisphere in spite of many NRG1-expressing cells (Fig 3A). Ipsilateral ischemic brain tissues transplanted with NRG1-AdMSCs showed a significant increase in NRG1 expression when assessed by ELISA (Fig 3B, F(2,6) = 9.88, p = 0.01, n = 3 per group). In order to further identify the source of increased NRG1 expression after NRG1-AdMSCs transplantation, western blot analysis was conducted (Fig 3C). Since we introduced short EGF-like domain of NRG1 to adenovirus, adenoviral NRG1 could be identified by 7 kD of molecular weight, which was only expressed in NRG1-infected AdMSCs (Fig 3C). Interestingly, in addition to exogenous NRG1 expression, endogenous NRG1 (35 kD) was also increased in NRG1-AdMSCs (Fig 3C and 3D). Relative quantitative analysis of both endogenous and exogenous NRG1 expression based on the value of PBS group revealed that transplantation of NRG1-AdMSCs (n = 6) significantly upregulated NRG1 compared to AdMSCs administration (n = 4) (t(8) = 3.61, p < 0.01 for endogenous NRG1, t(8) = 3.92, p < 0.01 for exogenous NRG1; Fig 3D). These results suggest that NRG1-AdMSCs can release adenoviral NRG1, stimulating endogenous NRG1 upregulation in the ischemic hemisphere.

### Transplantation of NRG1-expressing AdMSCs activates MAPK and Akt, with the upregulation of ErbB4 expression

**Fig 4:**
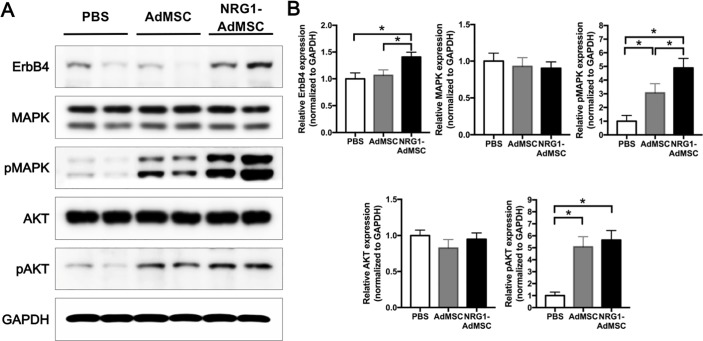
Transplantation of NRG1-AdMSCs activates ErbB4-associated downstream signaling pathways. (A) Representative western blot of three independent experiments results of ErbB4, MAPK, pMAPK, Akt, and pAkt. (B) Graphs showing relative expression levels of ErbB4, MAPK, pMAPK, AKT, and pAKT among PBS-, AdMSCs-, and NRG1-AdMSCs-treated groups. Expression of ErbB4, a receptor of NRG1, was markedly increased by NRG1-AdMSCs treatment (PBS, n = 7; AdMSCs, n = 8; NRG1-AdMSCs, n = 8). Moreover, pMAPK and pAkt expressions were significantly increased by NRG1-AdMSCs transplantation, compared to PBS-treated controls (PBS, n = 7; AdMSCs, n = 10; NRG1-AdMSCs, n = 10). *p < 0.05 by one-way ANOVA followed by LSD post hoc test. The data are presented as the mean ± SEM.

We then sought to explore the underlying molecular mechanisms of beneficial effects of NRG1-AdMSCs after ischemic stroke. To do that, we performed western blot to investigate NRG1-associated downstream signaling pathways. Despite there was no difference in the expression of the total MAPK and Akt among the groups treated with PBS, AdMSCs, and NRG1-AdMSCs, phosphorylated MAPK and Akt were markedly increased in the ischemic hemisphere injected with NRG1-AdMSCs (Fig 4, F(2,24) = 8.31, p < 0.01 for pMAPK, F(2,24) = 9.67, p < 0.01 for pAKT, n = 7 (PBS), n = 10 (AdMSCs), n = 10 (NRG1-AdMSCs)), demonstrating activated MAPK and Akt pathways by NRG1-AdMSCs transplantation. Moreover, at 14 d after MCAo, ErbB4, a receptor for NRG1, was significantly increased in NRG1-AdMSCs-treated group (Fig 4, F(2,20) = 4.80, p = 0.02, n = 7 (PBS), n = 8 (AdMSCs), n = 8 (NRG1-AdMSCs)). Collectively, our findings suggest that NRG1-AdMSCs transplantation after ischemic stroke can activate MAPK and Akt pathways, possibly through the upregulation of the NRG1 receptor, ErbB4.

## Discussion

In the present study, we demonstrated therapeutic potentials of AdMSCs engineered to produce NRG1 in ischemic stroke. We first confirmed that NRG1-AdMSCs could release higher levels of NRG1 compared to AdMSCs. Transplantation of NRG1-expressing AdMSCs into the ipsilateral striatum after ischemic stroke significantly reduced the brain infarct size and enhanced functional recovery, which was accompanied with the upregulation of both exogenous and endogenous NRG1 expression. Moreover, a NRG1 receptor, ErbB4, was upregulated in the ischemic hemisphere by NRG1-AdMSCs administration, in addition to the increased pMAPK and pAkt, crucial downstream molecules activated by NRG1-ErbB4 stimulation.

Therapeutic efficacies of transplanted AdMSCs after ischemic stroke have been demonstrated in numerous studies [35]. Consistent with previous reports, we also demonstrated functional improvements by AdMSCs administration. However, in spite of the significant amelioration of stroke-induced motor dysfunction, infarct size was not affected by AdMSC transplantation in our study, which is also shown in a couple of reports [36, 37]. AdMSCs-mediated functional improvements in ischemic stroke had been thought to be attributed to their subsequent differentiation into parenchymal cells replacing damaged tissues [7]. However, many studies later reported that MSCs could release trophic factors and other signaling molecules such as brain-derived neurotrophic factor (BDNF), vascular endothelial growth factors (VEGF), and transforming growth factor (TGF) β, contributing to the functional recovery following stroke [38–40]. Moreover, the capacity of MSCs to release neurotrophins and growth factors was markedly enhanced when MSCs were exposed to MCAo-subjected tissue homogenates [41], suggesting active interactions between MSCs and endogenous ischemic brain tissues. In line with these reports, secretome analysis of MSCs stimulated by TGFα revealed that NRG1 was one of the most enriched genes [42], implying that NRG1 may be responsible for the paracrine actions of MSCs following stroke. However, given the limitations of current stem cell therapy that cannot guarantee complete reversal of stroke-induced motor dysfunction, boosting the production of important growth factors in MSCs can be an attractive option to enhance therapeutic potential of MSCs. To prove this hypothesis, researchers have tried to upregulate various trophic factors such as BDNF, VEGF, glial cell-derived neurotrophic factor (GDNF), hepatocyte growth factor (HGF), placental growth factor (PIGF), and fibroblast growth factor 2 (FGF2) in human MSCs and found functional improvement after stroke, compared to naïve MSCs [33, 38–40, 43–47]. In accordance with prior reports, our NRG1-expressing AdMSCs, which was confirmed to release high levels of NRG1, could result in improved post-stroke motor function. Collectively, these data including our findings suggest that gene-modified stem cell therapy can be a useful, new approach for the treatment of ischemic stroke.

Interestingly, our NRG1-AdMSCs transplantation markedly elevated endogenous NRG1 expression in the ischemic cerebral issues, not to mention adenoviral NRG1 secretion. Since NRG1 itself can prevent stroke-induced pro-inflammatory and stress gene expressions, promoting neuronal survival against ischemic stroke [24, 48, 49], NRG1-AdMSCs-mediated high NRG1 expression during the critical post-ischemic period, together with beneficial roles of AdMSCs, may lead to better functional recovery and reduction in infarct volume, compared to adenoviral NRG1-, AdMSCs- or PBS-treated groups. It is also noteworthy that with the addition of NRG1-secreting AdMSCs, host ischemic brain tissues increased the endogenous NRG1 production, along with the NRG1 receptor, ErbB4, suggesting an enhanced NRG1-ErbB4-NRG1 autocrine loop by the transplantation of NRG1-AdMSCs. In support of this hypothesis, ischemic stroke can induce ErbB4 expression in neurons [50] and NRG1 ligand, possibly secreted from neuronal presynaptic terminals can positively regulate ErbB4 signaling [51]. Moreover, there is another report showing that ErbB4-exrpessing MSCs could increase NRG1 production and secretion, supporting positive feedback relationships of NRG1-ErbB4 signaling.

At the molecular level, NRG1 can activate multiple intracellular signal transduction pathways through interactions with ErbB receptor tyrosine kinases [16–18]. Our study demonstrated significant upregulations of phosphorylated MAPK and Akt in the ischemic brain of NRG1-AdMSCs-treated rats, with the increase of ErbB4 expression. Supporting our findings, activation of NRG1-ErbB4 signaling was neuroprotective, which was mediated by enhancing GABAergic transmissions in ischemic brain injury and schizophrenia [52–54]. These data suggest that ErbB4 activation can be crucial in NRG1-associated protective effects. With regard to downstream signaling pathways, ErbB4 is known to be linked to Ras-MAPK and phosphoinositide 3-kinase (PI3K)-Akt pathways [55]. In accordance with our findings showing increased pMAPK and pAkt by NRG1-AdMSCs transplantation, increased NRG1 expression by lipopolysaccharide-induced neuroinflammation could alter phosphorylation of Akt in the mouse brain [56]. Moreover, administration of NRG1β to autonomic ganglion neurons could also increase the expressions of pAkt and pMAPK [57], in line with our results. Interestingly, when compared to AdMSCs, transplantation of NRG1-AdMSCs significantly increased pMAPK expression in the ischemic hemisphere, while pAKT expression was upregulated in both AdMSCs- and NRG1-AdMSCs-treated groups. These data suggest that enhanced MAPK signaling can play a role in additional functional benefits of NRG1-AdMSCs compared to AdMSCs, whereas AKT signaling can serve to promote AdMSCs-mediated functional improvements after ischemic stroke. Further studies exploring differential molecular mechanisms of AdMSCs and NRG1-AdMSCs after ischemic stroke are warranted.

## Conclusion

Cell-based therapies have emerged as a new approach for the treatment of ischemic stroke. Various trophic factor-modified MSCs could enhance therapeutic potentials in the ischemic stroke by boosting secretory function of stem cells. In this study, we showed that transplantation of NRG1-expressing AdMSCs could significantly improve neurologic outcomes against MCAo-induced ischemic brain injury, compared to naïve AdMSCs or NRG1 overexpression. We also demonstrated that neuroprotective effects of NRG1-AdMSCs administration could be mediated by the upregulation of ErbB4. Finally, transplantation of NRG1-AdMSCs significantly increased the expression of pMAPK and pAkt compared to PBS-injected controls, suggesting the activation of MAPK and AKT signaling. As neuroprotective mechanisms of gene-modified MSCs transplantation for treating stroke are complex and are still not fully understood, additional investigations are necessary for deciphering complicated repair processes after ischemic brain injury.

## Supporting information

S1 File The ARRIVE guidelines checklist. (PDF)
